# Oral findings in Williams-Beuren syndrome

**DOI:** 10.4317/medoral.21834

**Published:** 2017-12-24

**Authors:** Shirlene-Barbosa-Pimentel Ferreira, Melissa-Machado Viana, Naiara-Gonçalves-Fonseca Maia, Letícia-Lima Leão, Renato-Assis Machado, Ricardo-Della Coletta, Marcos-José-Burle de Aguiar, Hercílio Martelli-Júnior

**Affiliations:** 1 Health Science Program, State University of Montes Claros, Unimontes, Minas Gerais State, Brazil; 2 Clinical Genetic Service, Federal University of Minas Gerais, UFMG, Belo Horizonte, Minas Gerais State, Brazil; 3 Department of Oral Diagnosis, Piracicaba Dental School - FOP/Unicamp, Piracicaba, São Paulo, Brazil; 4 Stomatology Clinic, Dental School, State University of Montes Claros, Unimontes, Montes Claros, Minas Gerais State, Brazil

## Abstract

**Background:**

Williams-Beuren syndrome (WBS; OMIM #194050) is a developmental disorder characterized by congenital heart disease, intellectual disability, dysmorphic facial features and ophthalmologic abnormalities. Oral abnormalities are also described in clinical manifestations of the disease. This paper describes orofacial features in patients with WBS.

**Material and Methods:**

Seventeen patients with a confirmed molecular diagnosis of WBS were examined for oral abnormalities through clinical oral evaluations and panoramic radiography.

**Results:**

Malocclusion, specifically with dental midline deviation, and high-arched palate were the most common findings.

**Conclusions:**

The present results contribute to knowledge on the orofacial manifestations of WBS. Since such patients with WBS may develop severe oral abnormalities, early detection and treatment can help improve their quality of life.

** Key words:**Congenital abnormalities, Orofacial features, Williams-Beuren syndrome.

## Introduction

Williams-Beuren syndrome (WBS; OMIM #194050) is a genetic disorder caused by the hemizygous deletion of 26 to 28 contiguous genes, including elastin (ELN) and LIM domain kinase 1 (LIMK1) on chromosome 7q11.23 (1). The prevalence is estimated to be one in every 7,500 live births (2). WBS leads to multi-systemic alterations. The classic form involves dysmorphic facial features, a unique personality and cognitive profile, congenital cardiovascular disease, intellectual disability and infantile hypercalcemia (3,4). Virtually every organ and system can be affected in WBS as a result of haploinsufficiency (4). Most cases are sporadic, although familial cases with autosomal dominant inheritance have been reported (5).

Despite the consistency of the overall clinical features, the broad spectrum of anomalies and phenotypic variability (within a population and in different populations) frequently undermine the clinical diagnosis (6,7). Several laboratory techniques are used to confirm WBS clinical suspicion. Fluorescent in situ hybridization is widely used for the molecular diagnosis of this syndrome (4). Multiplex ligation-dependent probe amplification, microsatellite DNA markers and array comparative genomic hybridization may also be employed (7).

Numerous clinical features are described in patients with WBS. In the oral cavity, wide mouth, malocclusion, micrognathism, enamel hypoplasia, excessive interdental space, hypodontia, microdontia and abnormal tooth morphology are among the wide spectrum of abnormalities identified in this disease (8,9). The aim of this report is to describe our experience in identifying orofacial features in Brazilian patients with confirmed diagnosis of WBS.

## Material and Methods

Seventeen patients diagnosed with WBS were examined. All patients were recruited from the Clinical Genetic Service of the Federal University of Minas Gerais (Belo Horizonte, Brazil), which is a specialized outpatient clinic for the diagnosis and follow up of patients with genetic disorders. In all cases, WBS was determined based on a molecular diagnosis.

All patients included in this study were submitted to a clinical evaluation, which included general and oral examinations. Radiographic images were also used to detect oral abnormalities. This study received approval from the Human Research Ethics Committee of the university. A signed statement of informed consent was obtained from all participants or their legal guardians, in the case of those under the age of 18 years.

## Results

**Figure 1:**
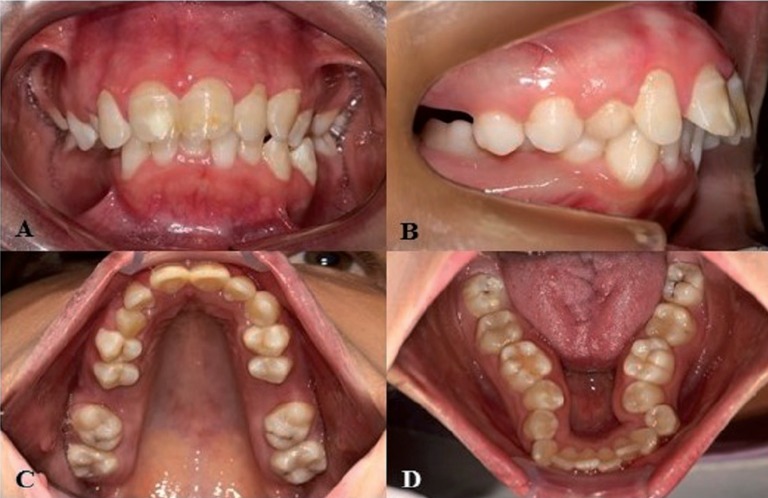
Oral findings in patient #15. (A) Malocclusion, dental midline deviation, (B) protruding maxillary teeth, mandibular retrognathism, maxillary prognathism, enamel hypoplasia, (C) high-arched palate, incisors with abnormal tooth shape, (D) irregular format of lower dental arch and tooth crowding.

**Figure 2:**
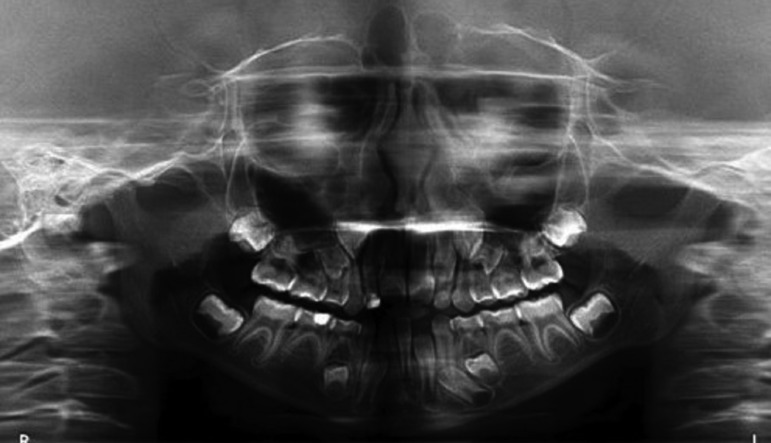
Panoramic radiograph of patient #4 of this study. Agenesis of the permanent maxillary and mandibular second premolars and permanent mandibular lateral incisor was observed.

The median age of the patients was 16 years (range: two to 36 years). Eleven patients were female and six were male.  Table 1 lists the main oral findings of the patients with WBS. All patients had malocclusion, except patient #1. Eleven patients (64.7%) exhibited dental midline deviation, and nine patients (52.9%) had a high-arched palate. Mandibular retrognathism was found in six patients (35.3%) and the dental arches had an irregular shape in three (17.6%) patients (Fig. 1A-D). Two patients (11.8%) exhibited tooth crowding and three (17.6%) exhibited excessive interdental spacing. Agenesis was found in three patients (17.6%) (Fig. 2).

**Table 1:**
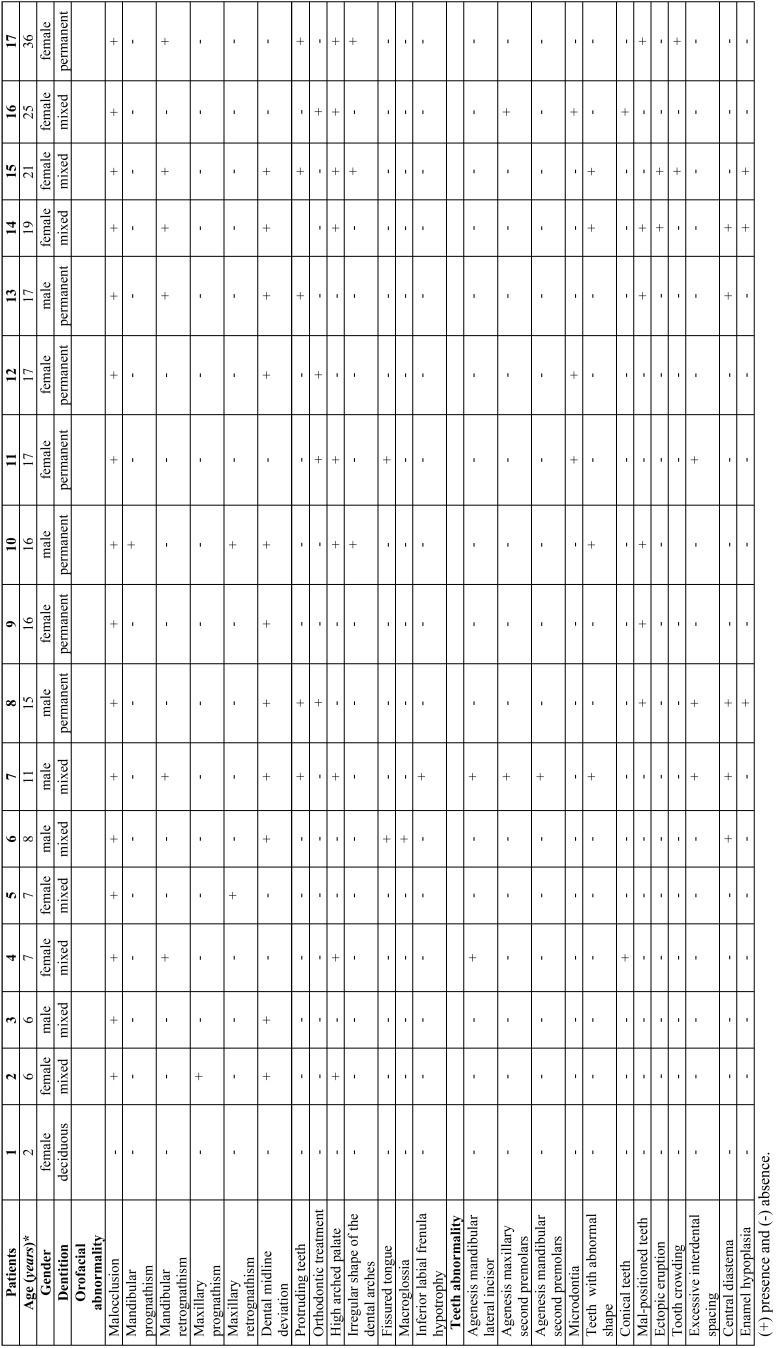
Orofacial aspects of the 17 patients with Williams-Beuren syndrome.

**Figure 3:**
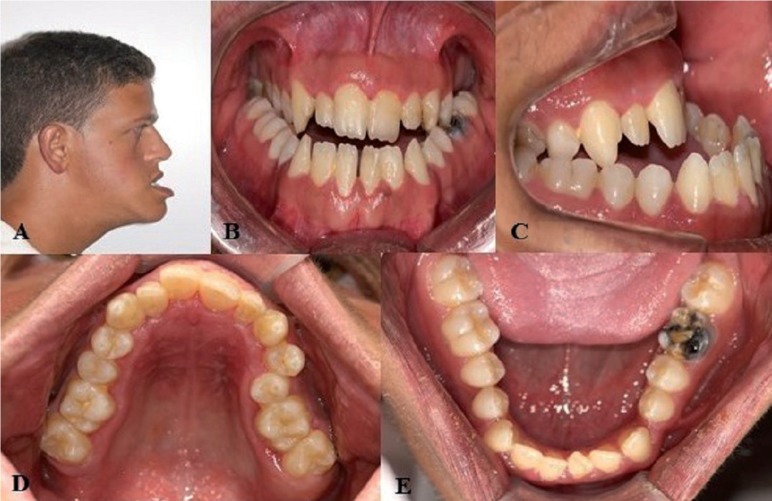
(A) Lateral view of patient #10 showing mandibular prognathism; (B) permanent dentition, malocclusion, dental midline deviation, (C) excessive mandibular prognathism, maxillary retrognathism, abnormal shape of upper canines, (D) high-arched palate, irregular format of upper dental arch and (D, E) malpositioned teeth.

Microdontia was found in three patients (17.6%) and maxillary retrognathism was found in two (11.8%) of the examined patients. Conical teeth were found in two patients (11.8%) and fissured tongue was found in two patients (11.8%). One patient exhibited maxillary retrognathism and mandibular prognathism (Fig. 3 A-C). Among the additional findings, inferior labial frenula hypotrophy was found in one patient (5.9%) and macroglossia was found in one patient (5.9%).

Specific types of malocclusion were observed in evaluated patients. Anterior open bite was showed in four (23.5%) patients, “Top-to-end” bite was observed in two (11.8%) patients, while anterior cross bite was detected in one (5.9%) patient. One patient (5.9%) showed anterior and posterior cross bite, and equally one (5.9%) patient had class III malocclusion.

## Discussion

WBS is a multi-systemic disorder characterized by developmental and physical abnormalities (11,12). Although facial dysmorphisms and dental anomalies are common in patients with WBS (8,11,13,14), the oral abnormalities are considerably varied and have received little attention in the literature (14-17). Thus, the present study was conducted with the purpose of describing orofacial characteristics found in this syndrome.

Our patients were submitted to oral examination in a Clinical Genetic Service, and no screening of WBS patients in a dental center was done for this study. Therefore, our random sample was heterogeneous. There were patients of different ages, and the findings have no correlation with the age range. Although, this sample aspect allowed us to identify a great diversity of orofacial alterations in evaluated patients. Here, only a 2-years-old female child did not exhibit oral abnormalities.

Oral abnormalities in patients with WBS were systematically investigated in the present study. All the anomalies were recorded and then compared with those reported in WBS. The most frequent finding was malocclusion. This alteration was present in 16 patients (94.12%), while dental midline deviation and high-arched palate was detected in 11 and 9 patients, respectively. Indeed, malocclusion is one of the most frequent common oral problems among the morphological and functional disorders of the orofacial complex in patients with WBS (14,15,18).

Anterior inclination of the maxilla, abnormal size, shape and number of teeth and enamel hypoplasia have been described in WBS patients (15-17). Additional oral alterations have also been reported, such as invagination of the maxillary incisors, small, slender roots, pulp stones, excessive interdental spacing, high prevalence of dental caries (8) and malocclusion (8,13-15).

A recent study reported oral findings in a patient with WBS. The intraoral examination revealed anterior open bite, excessive interdental spacing, enamel hypoplasia, dental caries, agenesis of the mandibular right second premolar and eruption chronology compatible with the age of the patient (14). Among other aspects reported in the present study, agenesis of the maxillary and mandibular second premolar and mandibular lateral incisor, widespread excessive interdental spacing and central diastema (described herein as excessive interdental spacing between the upper central incisors) were found.

WBS is characterized by typical dysmorphic facial features, such as full, prominent cheeks, a wide mouth, prominent lips and a long philtrum (11,12,15). The craniofacial abnormalities described in patients with WBS include anterior inclination of the maxilla, high mandibular plane angle, deficient chin bone (15,16) and micrognathia (9). In the present study, three patients had dental arches with an irregular shape. While craniofacial abnormalities are commonly reported in patients with WBS, no previous studies in the literature have described abnormalities in the shape of the dental arches in this population.

Although an orthodontic evaluation was not the purpose of this study, abnormal maxilla and mandible spatial positioning was found and the subjective morphological analysis of the profiles (19) revealed either protrusion or retrusion of the maxilla or/and mandible among the patients examined.

WBS is the result of deletion encompassing the ELN gene at 7q11.23 (11), with 26 to 28 genes usually identified within the deleted region (20,21). Studies have suggested that three members of the TFII-I gene family (Gtf2i, Gtf2ird1 and Gtf2ird2), which are expressed during odontogenesis, are potential candidate genes within the deleted region responsible for the tooth anomalies found in WBS (20,21). Gtf2ird1 may be associated with hypoplasia of the mandible, in addition to other craniofacial defects and dental alterations in individuals with WBS (21).

## Conclusions

The present study was found a large number of orofacial abnormalities in patients with WBS. The findings contribute to the knowledge on the oral manifestations of the syndrome. Therefore, such individuals should be submitted to dental evaluations to enable early detection and treatment, which could help improve their quality of life. All patients and their families in this study received genetic counseling and dental treatment.
